# Evaluation of a new transcutaneous bilirubinometer in newborn infants

**DOI:** 10.1038/s41598-022-09788-4

**Published:** 2022-04-07

**Authors:** Mikael Norman, Hüseyin Aytug, Hasan Basri Celebi

**Affiliations:** 1 Division of Pediatrics, Department of Clinical Science, Intervention and Technology, Karolinska Institutet, Novum, Blickagången 6A, 141 57 Stockholm, Sweden; 2 Department of Neonatal Medicine, Karolinska University Hospital, Stockholm, Sweden; 3 JAISY Health AB, Stockholm, Sweden; 4 School of Electrical Engineering and Computer Science, Division of Information Science and Engineering, KTH Royal Institute of Technology, Stockholm, Sweden

**Keywords:** Neonatology, Optical spectroscopy

## Abstract

To avoid brain damage in newborn infants, effective tools for prevention of excessive neonatal hyperbilirubinemia are needed. The objective of this study was to evaluate a new transcutaneous bilirubinometer (JAISY). For this purpose, 930 bilirubin measurements were performed in 141 newborn infants born near-term or at term (gestational age 35–41 weeks; postnatal age 1–6 days; 71 boys; including 29 infants with darker skin) and compared to those of a previously validated instrument (JM105). In each infant, the mean of three repeated measurements in the forehead was calculated for each instrument, followed by a similar measurement on the chest. The bilirubin values varied between 0 and 320 µmol/l (0–18.8 mg/dl). There was a high degree of agreement with significant correlations between bilirubin values measured with the two devices on the forehead (Pearson’s r = 0.94, *p* < 0.001) and the chest (r = 0.94, *p* < 0.001). The correlations remained after stratifying the data by gestational age, postnatal age and skin color. The coefficient of variation for repeated bilirubin measurements was 8.8% for JAISY and 8.0% for JM105 (*p* = 0.79). In conclusion, JAISY provides accurate and reproducible information on low to moderately high bilirubin levels in newborn infants born near-term or at term.

## Introduction

Neonatal jaundice is common and results in the clear majority from a transient and physiological accumulation of bilirubin. In a small proportion of infants, however, neonatal hyperbilirubinemia may become excessive which ultimately could cause brain damage^1–4^. Although a preventable condition, there have been some reports on increasing numbers of disabling neonatal hyperbilirubinemia^5,6^ and this condition is still a major cause of lifelong neurodevelopmental impairment with high costs for the affected family and the society^4,7^. While failed prevention may have different causes, inability to measure bilirubin, untimely measurements, and absence of repeated bilirubin measurements have been identified as important safety flaws^5^.

Guidelines for care of healthy newborn infants include screening and management of neonatal hyperbilirubinemia^8–14^. Several guidelines recommend that all infants have at least one bilirubin determination early after birth, and that the prognostic information of that bilirubin value in addition to known risk factors for neonatal hyperbilirubinemia^15,16^ should be used to determine when the next bilirubin measurement should take place. To avoid painful blood sampling and to have a quick result bedside, non-invasive, transcutaneous (t.c.) bilirubin determinations have reached widespread use as first-line screening tools in high-resource settings^17–19^.

Although judged as cost-effective^20^, t.c. bilirubin measurements in newborn infants are expensive^21^ which limits availability outside high-resource settings. Even within hospitals, there are usually only a few instruments. Given that many families nowadays are discharged home by the time neonatal hyperbilirubinemia peaks, there is an emerging need for accurate bilirubinometers for outpatient care and home monitoring. For this purpose, a new and simple t.c. bilirubinometer was developed. The objective of this study was to evaluate this device. The hypothesis tested was that the new bilirubinometer would be feasible for an intended use in near-term or term neonates, and that the test-device would perform as accurately as established standard instruments.

## Methods

The protocol of this study and an application was submitted and scrutinized by the Ethics Review Authority in Sweden before the study was executed. Because no personal information (no personal identities, dates or names) was collected and because t.c. bilirubin measurements were considered as part of the routine neonatal management, the Ethics Review Authority did not find that the project involved research according to §§3–4 of the Swedish Ethical Review Act, and that the study could be executed without a formal approval by the Authority. Informed consent for the measurements and data collection was obtained from all parents.

### Participants and setting

This study was executed at the delivery, maternity (the well-baby nursery) and neonatal units (in family rooms, no intensive care admissions were included) at Karolinska university hospital, Stockholm, Sweden between October and December 2020. Inclusion criteria were a gestational age of 35 weeks or more, a postnatal age less than 10 days, and newborn infants who had not received phototherapy or exchange transfusion for hyperbilirubinemia. Infants with major malformations or ongoing neonatal morbidity were not considered eligible for this study. All infants included in this study were tested for bilirubin levels as part of the clinical routine following the national Swedish guidelines issued in 2019^22^. Clinical characteristics of the mothers and their infants (n = 141) were collected to describe the study sample.

### Study protocol

The t.c. bilirubin measurements were performed by tester 1, a specialist neonatal nurse (n = 45) and by tester 2, a midwife (n = 96). Both testers were familiar with the DRÄGER jaundice meter (JM) 105 (Drägerwerk AG & Co, Lübeck, Germany) used in clinical routine care at Karolinska university hospital for t.c. bilirubin determinations. Tester 1 had a more thorough introduction to JAISY and was assisted by one of the authors (HBC) while using it, whereas tester 2 was more briefly introduced to JAISY after which she did all measurements on her own. Tester 1 performed measurements without any time pressure in the maternity units whereas tester 2 worked with families having scheduled follow-up appointments in which bilirubin screening was one of several items to attend to. The performance of tester 2—i.e., her ability to achieve reproducible measurements—was assessed after 70 measurements. This assessment was then presented to tester 2 before she continued the protocol.

Each infant had t.c. bilirubin levels determined at the forehead and at the chest. At each site, the mean of three subsequent t.c. bilirubin determinations was calculated for each instrument. In 14 of the 141 infants, a second test was performed.

The JM105 was considered gold standard and used according to the manufacturer's manual. The measuring principle of the JM105 has been described elsewhere^23^ and the instrument had been calibrated at the factory before use. According to the product information, the measurement range was 0–340 μmol/L (0.0–20.0 mg/dL) and the accuracy was ± 25.5 μmol/L (± 1.5 mg/dL) for infants > 35 weeks of gestational age^24^. The JM103—a precursor to the JM105—has shown good–excellent agreement and correlation (correlation coefficients varying between 0.77 and 0.96) with laboratory instruments for determination of serum bilirubin in newborn infants^25–28^.

**Figure 1:**
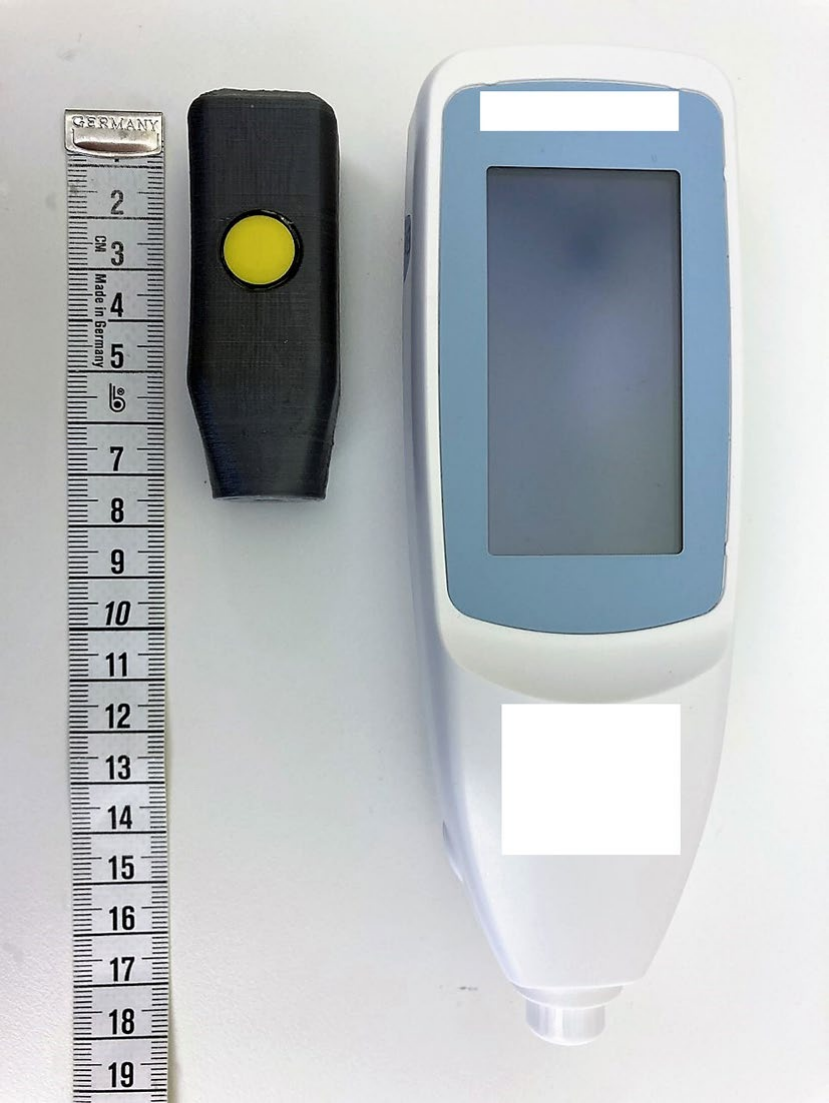
Photograph showing the two devices used in the study (JAISY to the left and JM105 to the right, measuring tape graded in centimeters).

A prototype of the new device JAISY was constructed. It consisted of a measuring probe with a size of 40 × 15 mm, powered by and connected to an ordinary cell phone. The measuring probe was held against the infant’s skin and a light flash was activated by a command on the cellphone screen. The light source was selected based on its wavelength range. Light absorption in bilirubin, hemoglobin, and melanin are wavelength dependent with characteristic variations in the visible spectrum range between 400 and 700 nm. Therefore, white and blue LEDs were chosen considering that they have suitable spectra. The emitted light was absorbed and reflected in the skin. The reflected light was detected and the spectrum of the reflected light was measured by photosensors placed at the tip of the probe. The reflected light was converted into electronic signals by the photosensor. JAISY determined the t.c. bilirubin level of the infant by photospectroscopy. The result could be displayed on the cellphone screen in μmol/L (or in mg/dl) but for the purpose of this study the measuring result was not displayed. Both devices are shown in Fig. 1.

The JAISY device was used in the same way as described for the JM105 with three consecutive measurements at each skin site. The forehead was in all infants measured before the chest, whereas the order of the measurements was randomly altered between the two instruments. The testers were blinded to the test result of JAISY and the instrument displayed only a 6-digit number which was noted in the study protocol. Every measured t.c. bilirubin value and the linked 6-digit number were automatically transmitted from the cellphone to a central database.

### Statistical analyses

For descriptive purposes, mean (standard deviation [SD]) values and numbers (proportions [%]) were used. To assess the validity of the new instrument, a mean bilirubin value was calculated from each set of three consecutive t.c. measurements per skin site and instrument. Based on these mean values, Pearsons´s correlation coefficients were calculated and Bland–Altman plots were constructed for t.c. bilirubin values using both methods. To assess reproducibility, the coefficient of variation (CV) for repeated measurements were calculated for each instrument and skin site, and for each tester. Chi-square test was used to test for differences between CVs. To describe associations between t.c. bilirubin measurements and gestational age, postnatal age, skin color and investigator, a series of stratified analyses were performed. All calculations were performed with the software Excel (Google drive) and Matlab R2020a.

## Results

Overall, 930 t.c. bilirubin measurements were performed in 141 infants. In 14 infants, a second measurement was performed. Clinical characteristics of the participants and their mothers are presented in Table 1.

**Table 1:** Characteristics of the participants.

Characteristics	N = 141
**Maternal and family history**	
Primipara	67 (47%)
Older sibling treated for neonatal hyperbilirubinemia	2/74 (2.7%)
Maternal blood group O	44/141 (31%)
**Mode of delivery**	
Vaginal, non-instrumental	94 (67%)
Vaginal, instrumental (vacuum extraction)	9 (6.4%)
Cesarean section	29 (21%)
*Missing*	9 (6.4%)
**Gestational age**	
35 weeks	4 (2.8%)
36 weeks	9 (6.4%)
37 weeks	7 (5.0%)
38 weeks	33 (23%)
39 weeks	37 (26%)
40 weeks	30 (21%)
41 weeks	18 (13%)
*Missing*	3 (2.1%)
**Postnatal age**	
1 day	10 (7.1%)
2 days	19 (13%)
3 days	23 (16%)
4 days	46 (33%)
5 days	24 (17%)
6 days	13 (9.2%)
*Missing*	6 (4.3%)
**Infant sex, girls**	70 (49.6%)
**Birth weight, grams**	3414 (514)
**Skin color**	
White	110 (78%)
Darker (brown or black)	28 (20%)
*Missing*	3 (2.1%)

Data are numbers (%) or mean (SD).

**Figure 2:**
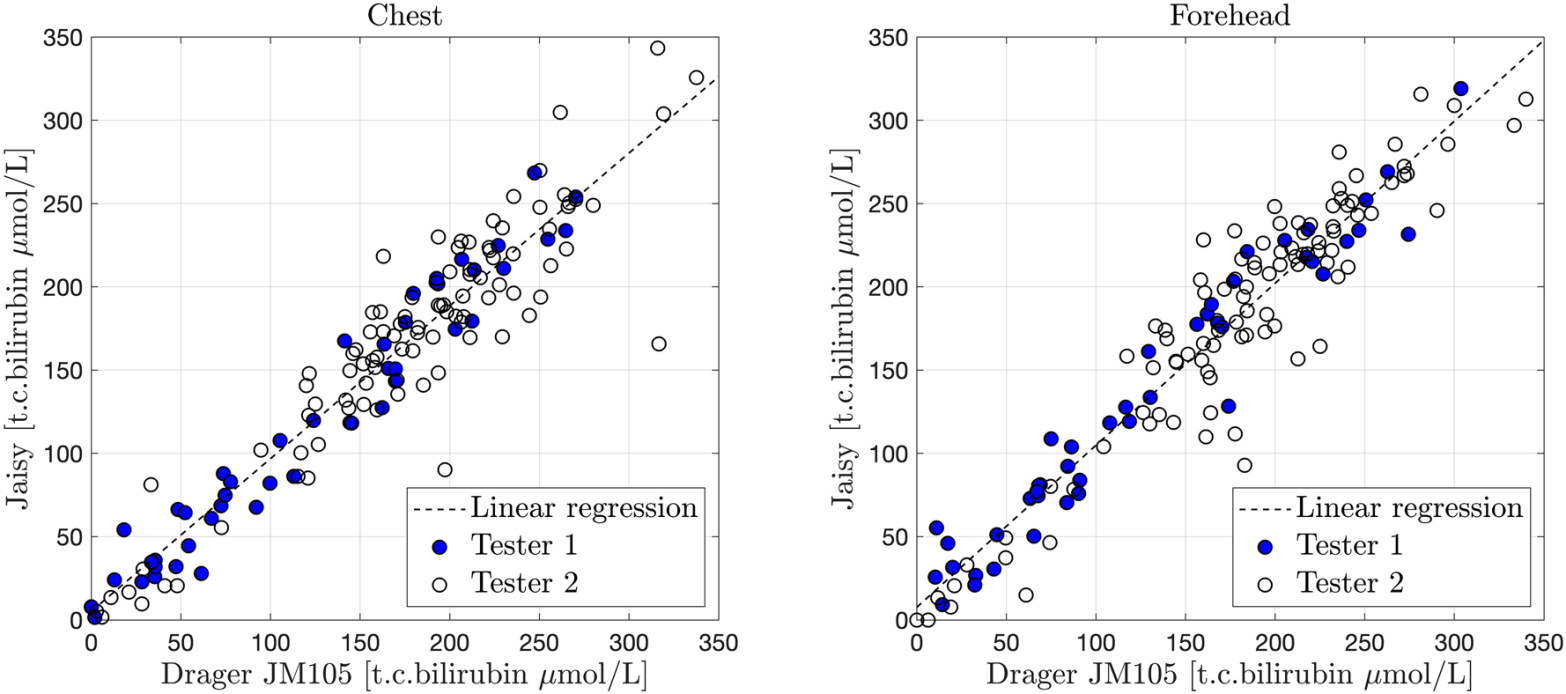
Correlation between t.c. bilirubin values (μmol/L) measured with JM105 and JAISY in the chest (β = 0.92, r = 0.94, *p* < 0.001) and forehead (β = 0.97, r = 0.94, *p* < 0.001) of 141 newborn infants (gestational age 35–41 weeks, postnatal age 1–6 days). Filled circles in blue = tester 1 and unfilled circles = tester 2.

**Figure 3:**
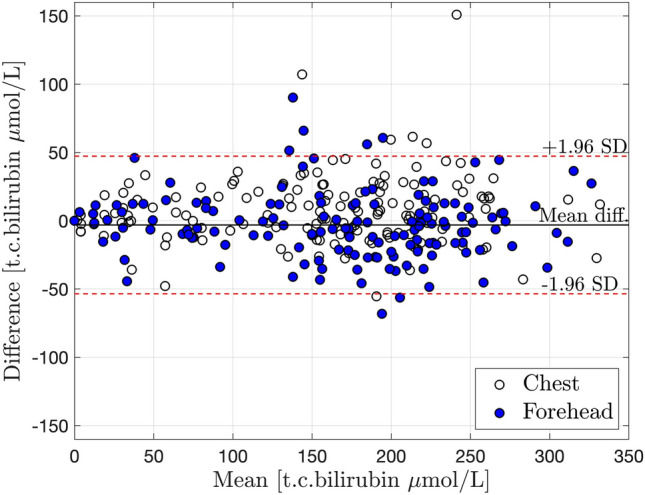
Bland–Altman plot with mean ± 2SD for an agreement between JM105 and JAISY measurements of t.c. bilirubin levels (μmol/L) in newborn infants. Unfilled circles = measurements at the chest, filled circles = measurements at the forehead. Mean = JM105 + JAISY/2. Difference = JM105 − JAISY.

The t.c. bilirubin measured with JM105 at the forehead varied between 0 and 302 μmol/L (0–17.8 mg/dL), and at the chest it varied between 0 and 310 μmol/L (0–18.2 mg/dL), i.e., all bilirubin values were within the measurement range of the instrument. There was a high correlation between t.c. bilirubin values assessed with JM105 and JAISY at the forehead (r = 0.94, *p* < 0.001) and the chest (r = 0.94, *p* < 0.001) with high degree of agreement (β = 0.97 at the forehead and 0.92 at the chest), Fig. 2. The Bland–Altman plot confirmed the agreement between the two instruments over the full range of t.c. bilirubin values, Fig. 3.

The overall mean (± SD) t.c. bilirubin concentrations using JM105 were 165 (± 79) μmol/L at the forehead and 161 (± 80) μmol/L at the chest. The corresponding bilirubin values for JAISY were 168 (± 81) and 153 (± 82) μmol/L, respectively. The mean differences (JM105 minus JAISY) in t.c bilirubin were − 3.1 (95% CI: − 7.2 to + 1.0) μmol/L at the forehead and 7.8 (95% CI 3.7–11.9) μmol/L at the chest.

The overall coefficient of variation (SD for within subject variation/overall mean value) for repeated measurements with JM105 was 12.8/165 = 7.7% at the forehead and 11.1/161 = 6.9% at the chest (*p* = 0.78 for difference between measuring site). The corresponding overall CVs for JAISY were 20/168 (11.9%) at the forehead and 21/153 (13.6%) at the chest (*p* = 0.67 for difference between sites).

Since there was no difference in CVs related to measuring site, measurements from forehead and chest were lumped together in a sub-analysis of reproducibility related to tester. The CVs for tester 1 (n = 45 babies, longer introduction for JAISY, assisted during measurements and no time pressure) were 9.9/126 = 7.8% for JM105 and 8.8/126 = 7.0% for JAISY (*p* = 0.81 for difference between instruments). The corresponding CVs for tester 2 working alone and under clinical conditions were for her first 70 babies 9.9/173 = 5.8% for JM105 and 25.7/167 = 15.3% for JAISY (*p* = 0.009 for difference). After discussing the performance with tester 2 and instructing her more carefully on how to hold the probe during the measurements to avoid inlet of ambient light, the CVs for her last measurements (n = 26 babies) were 15.4/177 = 8.7% for JM105 and 18.9/184 = 10.3% for JAISY (*p* = 0.64 for difference). Including only measurements obtained after proper education and instruction (n = 45 infants performed by tester 1 and last 26 infants by tester 2), there were no significant difference in reproducibility between the two instruments—the CV for repeated measurements was 12.3/154 = 8.0% for JM105 and the corresponding CV for JAISY was 13.6/154 = 8.8% (*p* = 0.79).

**Figure 4:**
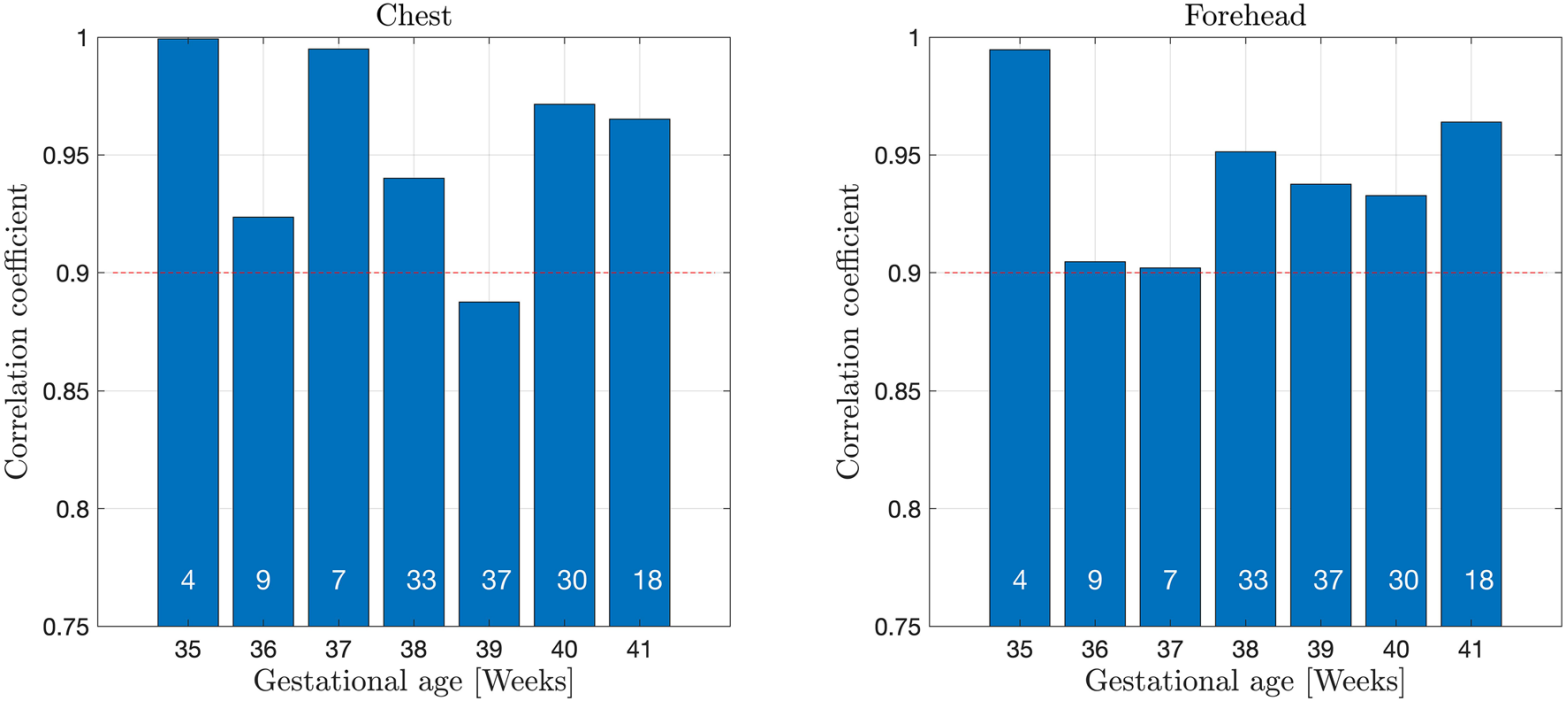
Correlation coefficients for t.c. bilirubin values measured with JM105 and JAISY at the forehead and chest, by gestational age. Numbers in each bar indicate the number of infants.

**Figure 5:**
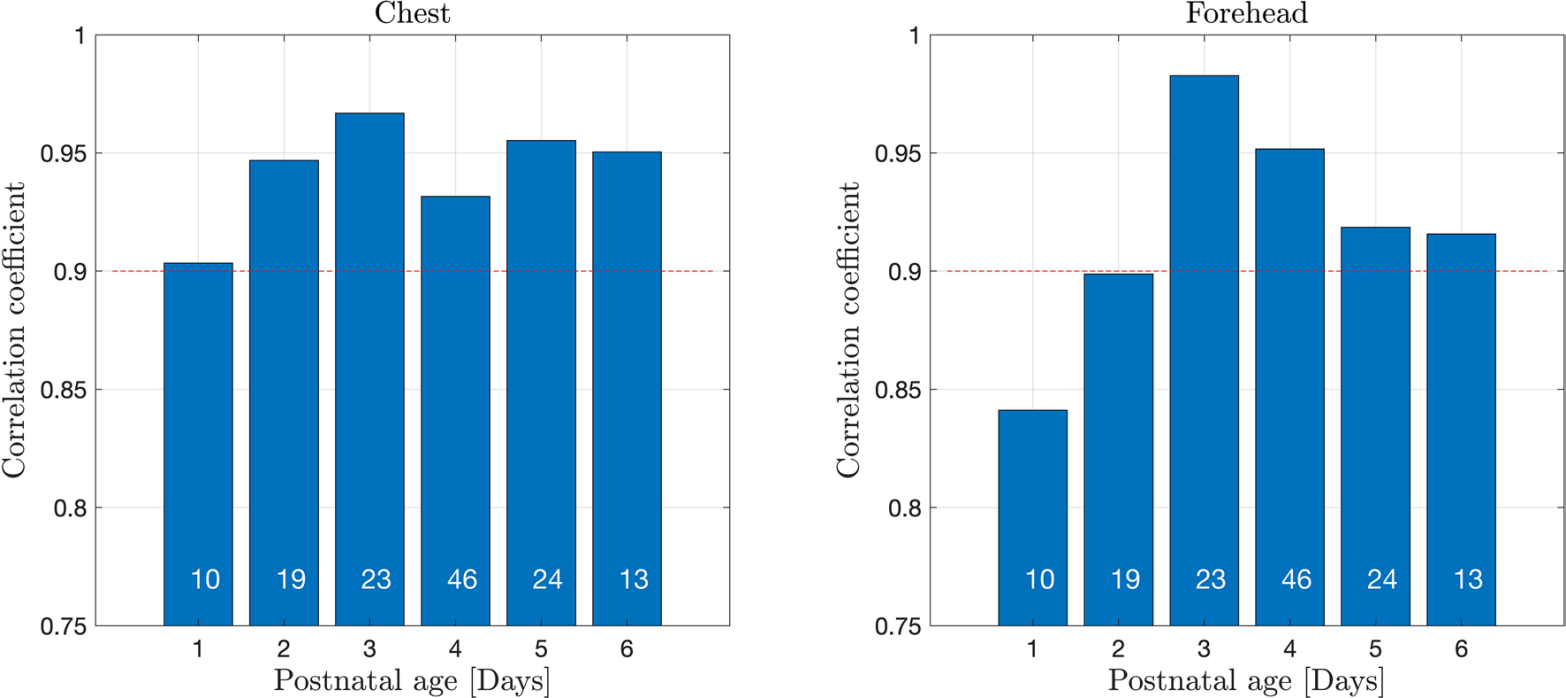
Correlation coefficients for t.c. bilirubin values measured with JM105 and JAISY in the forehead and chest, by postnatal age. Numbers in each bar indicate the number of infants.

The correlation coefficients between t.c. bilirubin values measured with JM105 and JAISY stratified by gestational age varied between 0.88 and 1.0, Fig. 4. The corresponding correlation coefficients between the two methods stratified by postnatal age varied between 0.84 and 0.98, Fig. 5. The correlation coefficient between t.c. bilirubin values measured with JM105 and JAISY in infants with white skin color was 0.95 at the forehead and 0.96 at the chest, and in infants with darker skin it was 0.92 at the forehead and 0.93 at the chest.

There were no adverse events during the measurements and a clear majority (n = 138/141 infants) were calm or sleeping during the measurements. Irrespective of device used, it took approximately 1 min to achieve three repeated measurements.

## Discussion

This study demonstrated that the JAISY device exhibited excellent agreement with a validated method used for determination of t.c. bilirubin in newborn infants. The reproducibility of JAISY was high and did not differ from that of JM105. JAISY was simple to use and feasible in neonates.

Irrespective of gestational age, the correlation coefficients between JM105 and JAISY were high. This suggests that JAISY can provide reliable information about bilirubin levels in term and near-term infants^25,26,28^ in line with national recommendations for use of t.c. bilirubinometers^8,14^.

The present findings support that t.c. bilirubin measurements are reliable for screening purposes on the first day of postnatal life^29^. Postnatal age did not significantly influence the methods agreement although on the first day of life, the correlation coefficients between bilirubin levels assessed with JM105 and JAISY were somewhat lower (r = 0.84–0.88) than in older infants. The most likely explanation for this finding is that bilirubin values were significantly lower on day 1 than in older infants with less accurate precision in both instruments at lower bilirubin values.

T.c. bilirubinometers—including JM103—has previously been reported to provide reliable estimates of bilirubin in infants with dark or brown skin^30,31^ although one report found lower agreement between serum and t.c. bilirubin values in people with darker skin than in those with lighter skin color^26^. Given the findings reported herein, JAISY can also be used in infants with different skin colors.

The methods agreement and reproducibility were unrelated to skin site, the forehead or the chest. This was expected because the two methods should provide a valid estimate of the same variable, i.e., the total serum-bilirubin, irrespective of skin site. Comparisons with total serum-bilirubin determinations have suggested that t.c. measurements at the chest are more reliable than at the forehead, especially 1–2 days after birth^31–33^. This study did not include serum-bilirubin values and cannot refute nor support those reports.

Similar to other t.c. bilirubin instruments, JAISY was shown to provide a simple and rapid way of obtaining an immediate result for bilirubin levels in neonates. The small size of the instrument makes wearing and disinfection easy and the connection to a standard cell phone enables data transfer to internal or external decision aids. These features should facilitate in- and outpatient bilirubin monitoring.

In order to estimate an impact of handling errors in a clinical situation in which staff in neonatal care would be introduced to the new instrument without much training, we introduced tester 2 more briefly than tester 1 (both testers were blinded to their reproducibility during measurements). We found that tester 2 initially performed less reproducible measurements than tester 1. Therefore and for proper use and reliable test results, users need clear handling instructions and some basic practice.

Although rare, 10/282 (3.5%) t.c. bilirubin values estimated by JAISY deviated 2SD (50 μmol/L) or more from JM105, and in 9 out of 10 of these deviations the bilirubin assessed with JAISY was lower than that assessed by JM105. The JAISY t.c. bilirubin values that were more than 2 SD below the mean were in 9/10 measurements assessed by tester 2 and before she had had feedback on performance. The most likely explanation was that ambient light had been diluting the reflected light from the skin because of suboptimal skin contact with the probe. After instruction on how to handle the probe, recommending tester 2 a closer skin contact to exclude incoming ambient light, the accuracy and reproducibility of tester 2 improved and became comparable to tester 1.

The strengths of this study include application of the JAISY instrument in a context of its intended use, i.e., in clinical practice with different users, including infants of different gestational and postnatal ages and with different skin colors. The users were blinded to the JAISY test-result which excluded assessment bias. A sufficiently large number of measurements was performed to cover the range of bilirubin levels commonly seen in newborn infants and to assess associations to gestational and postnatal age.

Limitations include selection of near-term and term infants only, whereas preterm infants were excluded. Moreover, the JAISY device was not evaluated in infants undergoing phototherapy for neonatal hyperbilirubinemia or with bilirubin values exceeding 310 μmol/L (18.2 mg/dL). The comparison of bilirubin values did not include blood test for determination of serum-bilirubin. Transcutaneous bilirubinometers should be considered as screening tools and critically high bilirubin values, as well as decisions on treatment of hyperbilirubinemia should always be validated by determinations of the concentration of total bilirubin in serum. However, determinations of t.c. bilirubin with JM103 have repeatedly been reported to exhibit good agreement with laboratory analyses of bilirubin from blood tests, at least up to 250 μmol/L (14.7 mg/dL)^25–28^. JAISY is not yet commercially available and pricing remains to be established after regulatory approvals, production and marketing expenditures have been taken into account.

In conclusion, JAISY provides accurate and reproducible information on low to moderate bilirubin levels in newborn infants born near-term or at term.

## Data Availability

The datasets generated and analyzed during the current study are available from the corresponding author on reasonable request.
